# Beliefs and attitudes about breast cancer and screening practices among Arab women living in Qatar: a cross-sectional study

**DOI:** 10.1186/1472-6874-13-49

**Published:** 2013-12-13

**Authors:** Tam Truong Donnelly, Al-Hareth Al Khater, Salha Bujassoum Al-Bader, Mohamed Ghaith Al Kuwari, Nabila Al-Meer, Mariam Malik, Rajvir Singh, Sofia Chaudhry, Tak Fung

**Affiliations:** 1 University of Calgary, 2500 University Dr. NW, Calgary, Alberta, T2N 1 N4, Canada; 2 Hamad Medical Corporation, Hamad General Hospital, P.O. Box 3050, Doha, Qatar; 3 Aspetar, P.O. Box 29222, Doha, Qatar; 4 Primary Health Care Corporation, P.O. Box 26555, Doha, Qatar; 5 University of Calgary – Qatar, Al Rayyan Campus, Al Forousiya Road, P.O. Box 23133, Doha, Qatar

**Keywords:** Qatari women, Breast cancer screening, Breast self-examination, Clinical breast examination, Mammogram, Arab women, Beliefs and attitudes, Breast cancer in the Middle East

## Abstract

**Background:**

Despite rising breast cancer incidence and mortality rates, breast cancer screening (BCS) rates among women in Qatar remain low. Previous studies indicate the need to better understand the many complex beliefs, values, and attitudes that influence Arab women’s health seeking behavior for the development of culturally appropriate and effective intervention strategies to address breast cancer in the Middle East. This study investigates beliefs, attitudes, and BCS practices of Arabic-speaking women in Qatar.

**Methods:**

A multicenter, cross-sectional quantitative survey of 1,063 (87.5% response rate) Arabic-speaking female Qatari citizens and non-Qatari residents, 35 years of age or older, was conducted in Qatar from March 2011 to July 2011. Associations between beliefs and BCS practice were estimated using chi-square tests and multivariate logistic regression analyses. Participants who adhered to BCS guidelines (BCS practice = Yes) were compared to those who did not (BCS practice = No).

**Results:**

In addition to low levels of awareness and low participation rates in BCS, one quarter of the participants stated their doctors talked to them about breast cancer, and less than half of the women interviewed believed breast cancer can be prevented. Women who engaged in BCS practice were more likely to have a doctor who talked to them about breast cancer, to believe they were in good–excellent health, that cancer can be prevented, or that cancer might be hereditary. The majority wanted to know if they had cancer and felt their health care needs were being met. The main reasons given for not planning BCS were lack of a doctor’s recommendation, fear, and embarrassment.

**Conclusions:**

These findings indicate that a variety of channels (health care providers, media, breast cancer survivors, community leaders) should be utilized to create culturally appropriate breast cancer intervention programs and increased awareness of breast cancer, BCS, and the benefits of early detection of breast cancer. Employment of these measures will reduce breast cancer mortality rates among Arabic-speaking women living in the State of Qatar.

## Background

Qatar, like many other Middle Eastern countries, comprises a traditional, collectivist society. H.H. Sheikha Moza bint Nasser, the most respected and celebrated woman of Qatar, once described Qatar as “a rising homeland that confidently embraces modernization and proudly observes tradition” (personal speech, Msheirab Enrichment Centre, Qatar). Thus, Qatar has the potential to offer the best of the West and the East by offering state-of-the-art medical technology while incorporating social, cultural, and religious principles to address the health care concerns of its two million people. The government of Qatar has made health care research as one of national research priorities; close to 3% of its annual GDP ($3.5 billion USD, more than any other country in the world) is allocated to funding research in Qatar. Therefore, health care programs and research aimed at addressing cancer in Qatar can significantly impact the Middle East region and Muslim and Arab women worldwide.

Breast cancer is one of the most commonly diagnosed and leading causes of cancer-related deaths among women just after lung cancer [1,2]. A 2011 Lancet study found that whereas breast cancer diagnoses occurred more frequently in developed countries in 1980, incidence rates have become greater in developing countries since 2010 [3]. From 2002 to 2020, breast cancer mortality rates are expected to rise at greater rates in developing countries than in developed countries [4]. In the Middle East, Arab women face a significantly higher risk of mortality because their cancer is often diagnosed at a late stage of the disease [5-10]. Among Qatari women, the leading cancer diagnosis, far greater than diagnosis of other cancers, is breast cancer [7,11].

When combined with appropriate treatment, early detection through breast cancer screening (BCS) activities that include breast self examination (BSE), clinical breast examination (CBE), and mammography, has been shown to decrease cancer mortality rates by 25–30% [12,13]. Studies of Arab women in the Middle East indicate low BCS participation rates [5,14-19]. A previous study conducted in Qatar found that 24.9% of the study population (women 30 to 55 years) practiced BSE regularly, 23.3% had undergone CBE, and 22.5% had undergone mammography [17].

A cancer diagnosis is often accompanied by social stigma in the Middle East, and limited physician disclosures of cancer diagnoses often coincide with social and cultural norms [19]. Because cultural beliefs, values, and attitudes have been found to influence perceptions of cancer and BCS practice [14,20,21], these factors must be investigated before a socially and culturally appropriate intervention strategy that addresses the threat of breast cancer in Qatar can be designed [6,14,22-24].

### Facilitators and barriers to breast cancer screening

Studies indicate that a BCS recommendation from a physician, family member, or friend; awareness of breast cancer and the importance of BCS; a family history of breast cancer; and a higher education level, residence in an urban area, employment, and higher socioeconomic status are important facilitators of BCS behavior [9,15-17,25,26].

Lack of a physician’s recommendation, fear of the BCS procedure and fear of finding cancer, low perceived risk of cancer or effectiveness of BCS, time, cost, preference for a female health professional, accessibility of the health care system, and embarrassment related to the BCS procedure are barriers to clinical breast examination and mammogram practice among Arab women [5,6,9,14,15,17]. In other studies, male relatives who objected to BCS were mentioned by study participants as a barrier to BCS (8.9% in Qatar and 2.7% in the United Arab Emirates) [15,17].

Despite fear of cancer and low levels of BCS participation, Arab women are eager to learn more about breast cancer and its screening activities [27-31]. To develop culturally appropriate awareness and effective intervention strategies to address breast cancer in Qatar, it is imperative that health care providers understand the many complex beliefs, values, and attitudes that influence Arab women’s health seeking behaviors with respect to breast cancer.

### Kleinman’s Explanatory model of health, disease, and illness

According to Kleinman’s Explanatory Model, “beliefs about sickness … including treatment expectations … affect the way individuals think about and react to sickness and choose among and evaluate the effectiveness of the health care practices available to them” [32]. Thus, individuals’ explanatory models are derived from their knowledge and values, which are informed by their specific sociocultural backgrounds. One of the major deterrents of client compliance, satisfaction, and appropriate use of health care services was the difference in explanatory models between recipients and providers of health care [32,33]. Thus, providing effective health care requires that providers be able to elicit and recognize recipients’ beliefs and values with respect to their understandings of illnesses and treatments, and to negotiate these differing perspectives [32]. The objective of this study was to gain information about Arab speaking women’s practice of breast cancer screening, and their knowledge, cultural beliefs, and values regarding breast cancer and its screening for early detection and treatment.

This paper reports (a) BCS participation rates of Arabic women living in Qatar, and (b) relationships between Arabic women’s BCS practice, their beliefs, values, and attitudes toward BCS, and selected sociodemographic factors. We hypothesize that there is a relationship between Arabic women’s beliefs, values, and attitudes toward BCS and their participation in BCS activities in Qatar.

## Methods

### Study population

Participant inclusion criteria included being 35 years or older (as previously recommended by national guidelines for BSE and CBE [23]), ability to speak Arabic, recruitment from one of seven designated hospitals and community health clinics in Qatar, and residence in Qatar for at least 10 years (to ensure the participant’s familiarity with Qatar’s social, cultural, and health care context). Based on Qatar’s 2010 census data, the study’s sample size was calculated using a 95% confidence level and Cochran’s formula for sample size [34,35]. To ensure representation of women living in various populated regions in Qatar, participants were recruited from hospital and health clinic settings in the capital of Qatar, south of Qatar, and north of Qatar [25]. It was not feasible to conduct a cross-sectional survey with randomly selected women participants because of our limited access to the female population in Qatar. Therefore, using a nonprobability convenient sampling technique, 1,215 self-identified Arabic women who met the study’s inclusion criteria were invited to participate in the survey; 1,063 women (40% more than the required sample size calculation using a margin of error of 3.5%) participated in a 30-minute face-to-face interview (87.5% response rate). High response rate was achieved as the result of highly trained female nurse interviewers, who were fluent in both Arabic and English; the interviewers gave thorough explanations of the study to participants and conducted face-to-face interviews in Arabic on site. To ensure diversity of participants and represent the general female Arab population, study participants were approached and interviewed in person during different days of the week and different times of the day [25].

Ethics approval for this research study was obtained from the Hamad Medical Corporation Research Committee (Ethics Approval Reference No: RC/1744/2010), the Qatar Supreme Council of Health (Ethics Assurance No: SCH-A-UCQ-050), and the University of Calgary’s Conjoint Health Research Ethics Board (Ethics ID: E-23551). Consent to participate in this study was obtained from each participant. Prior to conducting an interview, each participant was given an explanation of the study and informed of her rights according to the standard interview guideline. Participants were assured that all information would remain confidential and interview questionnaires were stripped of identifying information to preserve confidentiality. No incentive was given to participants of the survey.

### Questionnaire and data collection method

Data collection was obtained from interviews using a structured survey questionnaire. Interviews were conducted in Arabic by seven female nurses fluent in Arabic and English. Survey questionnaire items were incorporated from previous peer-reviewed surveys on breast cancer research in the United States and Australia with permission from authors, and further refined after a pilot study field-tested the questionnaire in Qatar [36-39]. Forward- and back-translations of the survey questionnaire into Arabic and English were carried out to ensure lexical equivalence.

### Statistical analysis

Descriptive statistics (mean, standard deviations for interval variables and frequency) and chi-squared tests were performed to determine associations between categorical dependents and categorical predictors. Simultaneous multivariate logistic regression analyses using the “Enter” method was used to further assess the association of preselected factors related to beliefs, values, attitudes, and sociodemographics with binary dependent variables (e.g., practice of BSE, CBE, and mammogram). Multicolinearity for all covariates significant after bivariate analyses was tested before using them in the multivariate logistic regression analyses. Participants assessed with appropriate adherence to BCS recommendations (BCS Practice = Yes) were compared to those who were not (BCS Practice = No). Statistical significance levels were established at alpha = 0.05 (only statistically significant predictors are reported in Table 1). Data analyses were conducted by and performed under direct instruction of two senior biostatisticians using SPSS version 20.

**Table 1:** Association between selected significant factors and appropriate BCS practice

			**Adjusted OR (95% CI)**	**P value**
**Predictors of BSE practice (n = 1061)**				
Health status (Wald χ^2^(1) = 6.87)				
Poor - Fair (reference)				
Good - Excellent			2.03 (1.20 – 3.44)	0.009*
Is there anything you can do to prevent cancer?				
No (reference)				
Yes			1.85 (1.29 – 2.67)	0.001*
Gender of HCP preference (Wald χ^2^(1) = 6.63)				
Male or no preference (reference)				
Female HCP			0.49 (0.29 – 0.85)	0.010*
Why do you think people get cancer – Cancer is hereditary?				
No (reference)				
Yes			1.68 (1.09 – 2.57)	0.018*
**Model summary**				
**−2 Log likelihood**	**Cox & Snell R Square**	**Nagelkerke R Square**		
**814.10**	**0.04**	**0.07**		
**Predictors of CBE practice (n = 1061)**				
Is there anything you can do to prevent cancer?				
No (reference)				
Yes			1.59 (1.21 – 2.10)	0.001*
Why do people get cancer – God’s punishment?				
No (reference)				
Yes			0.52 (0.33 – 0.83)	0.006*
Doctor is understandable				
No (reference)				
Yes			2.15 (1.55 – 2.98)	<0.001*
Gender of HCP preference (Wald χ^2^(1) = 6.46)				
Male or no preference (reference)				
Female HCP			0.56 (0.36 – 0.88)	0.011*
Why do you think people get cancer – Cancer is hereditary?				
No (reference)				
Yes			1.73 (1.27 – 2.36)	0.001*
**Model summary**				
**−2 Log likelihood**	**Cox & Snell R Square**	**Nagelkerke R Square**		
**1229.24**	**0.08**	**0.12**		
Is there anything you can do to prevent cancer?				
No (reference)				
Yes			1.59 (1.11 – 2.26)	0.011*
Why do people get cancer – God’s punishment?				
No (reference)				
Yes			0.51 (0.28 – 0.95)	0.035*
Doctor is understandable				
No (reference)				
Yes			1.81 (1.18 – 2.79)	0.007
Why do you think people get cancer – Cancer is hereditary?				
No (reference)				
Yes			1.68 (1.14 – 2.48)	0.009*
**Model summary**				
**−2 Log**				
**likelihood**	**Cox & Snell R Square**	**Nagelkerke R Square**		
**759.89**	**0.07**	**0.10**		
**Predictors for *not *** **planning a CBE within the next 12 months (n = 538)**				
Fear of knowing you might have cancer				
No (reference)				
Yes			0.43 (0.20 – 0.94)	0.034*
Embarrassment				
No (reference)				
Yes			0.32 (0.15 – 0.70)	0.004*
**Model summary**				
**−2 Log likelihood**	**Cox & Snell R Square**	**Nagelkerke R Square**		
**508.83**	**.06**	**0.10**		
**Predictors for *not *** **planning a Mammogram (40+ years old, n = 363)**				
Fear of knowing you might have cancer				
No (reference)				
Yes			0.09 (0.01 – 0.69)	0.021*
**Model summary**				
**−2 Log likelihood**	**Cox & Snell R Square**	**Nagelkerke R Square**		
**300.88**	**0.10**	**.16**		

*Significant at 0.05 level.

## Results

### Demographics

The study population was fairly homogenous in terms of marital status (78.9% married), religion (98.2% Muslim), and living area (88.7% in urban areas). Participants’ ages ranged from 35–82 years (*M* = 44.9, *SD* = 8.4), 52% were Qatari nationals, and 47.9% were non-Qatari residents (from the Levant, North Africa, neighboring Arab peninsular/GCC countries, or other countries in the greater Middle East), two-thirds had lived in Qatar for 30+ years, 33.3% had a university education, 36.6% of the married participants’ husbands had a university education, and 65.9% of the participants were unemployed (89.3% of whom were homemakers). Additional analyses found that younger participants were more likely to be university educated than older participants, and the eldest age group (50+ years) was made up of more Qatari nationals than non-Qataris (p < 0.05) (Table 2).

**Table 2:** Selected demographic characteristics of participants (N = 1,063)

**Characteristic**	**No. (%) of participants**
**Age (years)** (*M* = 44.9, *SD* = 8.4)*	
35-39	365 (34.4)
40-49	399 (37.6)
50+	297 (28.0)
**Nationality**	
Qatari citizen	554 (52.1)
Non-Qatari resident	509 (47.9)
**Marital status**	
Single	224 (21.1)
Married	839 (78.9)
**Religion**	
Muslim	1044 (98.2)
Christian	19 (1.8)
**Length of residence in Qatar (years)**	
10-29	332 (31.2)
30-49	551 (51.8)
50+	180 (16.9)
**Living area**	
Urban	943 (88.7)
Semi-urban	120 (11.3)
**Education level of participant**	
≤Primary/Intermediate	359 (33.8)
Secondary/Trade School	350 (32.9)
University	354 (33.3)
**Education level of participant’s husband (n = 896)**	
≤Primary/Intermediate	276 (30.8)
Secondary/Trade School	292 (32.6)
University	328 (36.6)
**Employment status of participant**	
Employed	362 (34.1)
Unemployed	701 (65.9)

*2 participants did not answer this question.

### BCS awareness and screening practice

**Figure 1:**
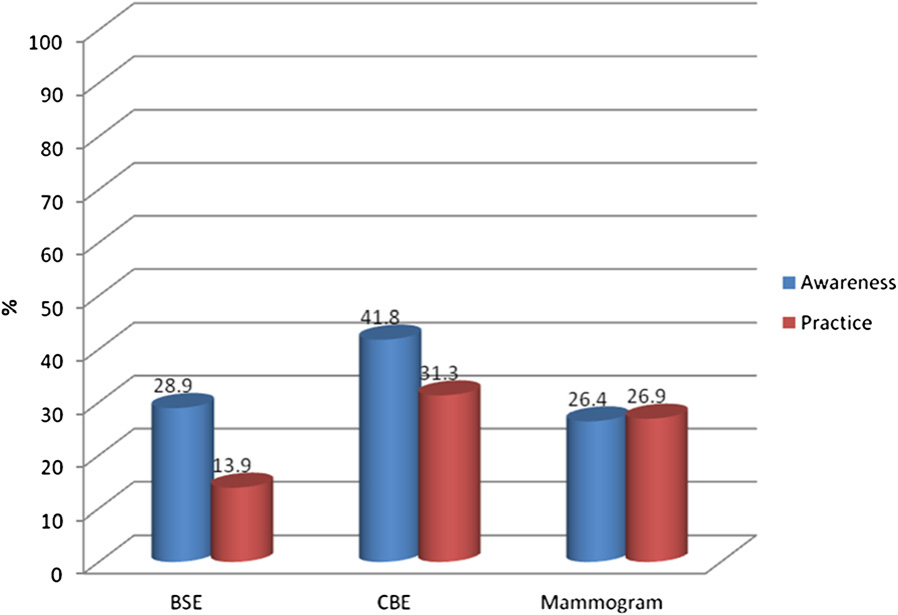
BCS Awareness and Practice (n = 1063).

Fewer than half of the participants were assessed with having awareness of the most recent BCS recommendations, and fewer than one third practiced BCS according to these recommendations. Additional analyses indicated that over 50% of the participants had never participated in a BCS activity. Participants’ previous BSE experience was significantly related to increased CBE and mammogram practice, and previous CBE experience was significantly related to increased mammogram practice (p < 0.05) (Figure 1).

### Beliefs and attitudes

The majority of participants stated that their health status was “good” or “excellent” (76.2%). When asked why people get cancer, participants responded “fate,” “having an unhealthy lifestyle,” “not breastfeeding one’s baby,” or “hereditary factors.” Less than half believed that cancer is preventable (42.8%), and less than one fifth reported that cancer was a punishment from God, bad luck, or that cancer was contagious.

The majority of participants reported that they trusted and understood their health care provider (HCP), they felt their health care providers respected them, and that their health care needs were being met. Although only 24.4% of the participants reported that their doctors had talked to them about breast cancer, most said they would make a mammogram appointment if they received a recommendation from their HCP. Most participants said they would want to know if they were diagnosed with cancer (86.6%), and preferred to consult doctors (72.4%) and other HCPs (90.8%) who were female; only 2.1% preferred that a nurse examine them rather than a doctor. Approximately half of the participants had no preference for their HCPs’ language (48.6%) (Table 3).

**Table 3:** Selected beliefs and attitudes of participants towards cancer, BCS and HCPs (N = 1,063)

**Variable**	**No. (%) of participants**
Health status*	
Poor – Fair	252 (23.8)
Good – Excellent	809 (76.2)
**Is there anything you can do to prevent cancer?**	
No/don’t know	608 (57.2)
Yes	455 (42.8)
**Would you want to know if you were diagnosed with cancer?**	
No/don’t know	142 (13.4)
Yes	921 (86.6)
**What type of HCP would you prefer?**	
Male HCP or no preference	98 (9.2)
Female HCP	965 (90.8)
Nurse or no preference*	293 (27.6)
Doctor	768 (72.4)
Non-Arab HCP or no preference	517 (48.6)
Arab HCP	546 (51.4)
**Why do people get cancer?**	
It’s God’s punishment	
No	914 (86.0)
Yes	149 (14.0)
It’s fate/destiny	
No	34 (3.2)
Yes	1029 (96.8)
It’s bad luck	
No	960 (90.3)
Yes	103 (9.7)
Cancer is contagious	
No	1016 (95.6)
Yes	47 (4.4)
Cancer is hereditary	
No	349 (32.8)
Yes	714 (67.2)
Unhealthy lifestyle	
No	71 (6.7)
Yes	992 (93.3)
Not breastfeeding their babies	
No	197 (18.5)
Yes	866 (81.5)
**Doctor has talked to participant about breast cancer**	
No	804 (75.6)
Yes	259 (24.4)
**Participant feels her healthcare needs are met**	
No	59 (5.6)
Yes	1004 (94.4)
**Participant trusts doctor**	
No	18 (1.7)
Yes	1044 (98.3)
**Participant feels she is treated respectfully by HCP**	
No	21 (2.0)
Yes	1042 (98.0)
**Doctor is understandable**	
No	357 (33.6)
Yes	706 (66.4)
**Participant would make mammogram appointment if HCP recommended**	
No	66 (6.2)
Yes	997 (93.8)
**Participant would make mammogram appointment if she received letter from HCP recommending it**	
No	439 (41.3)
Yes	624 (58.7)

*2 participants did not answer these questions.

**Figure 2:**
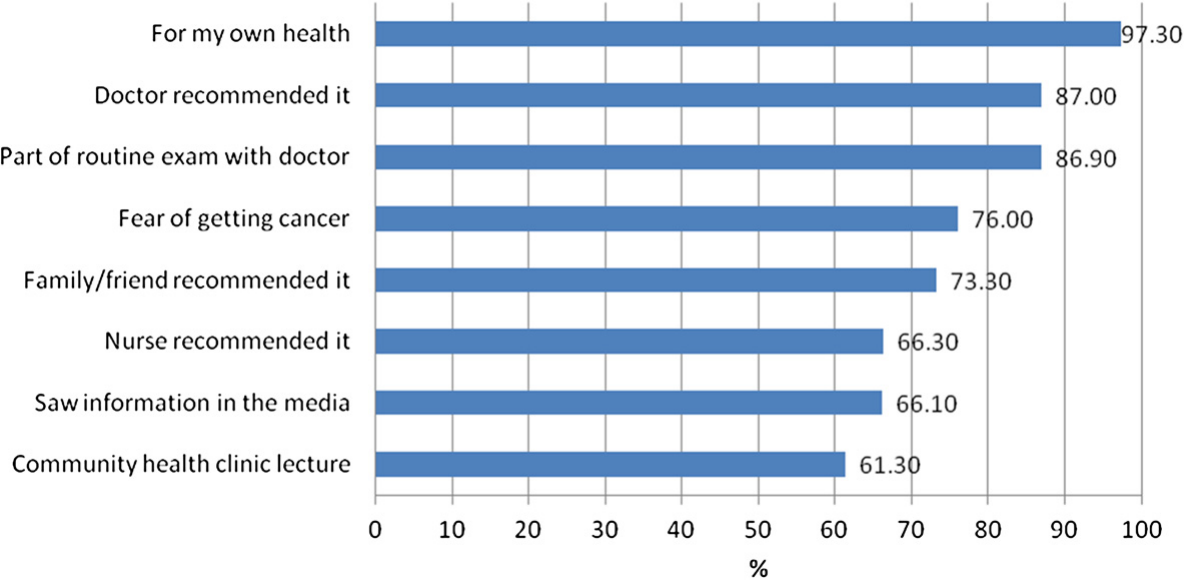
Reasons Participants are Planning CBE (n = 525).

**Figure 3:**
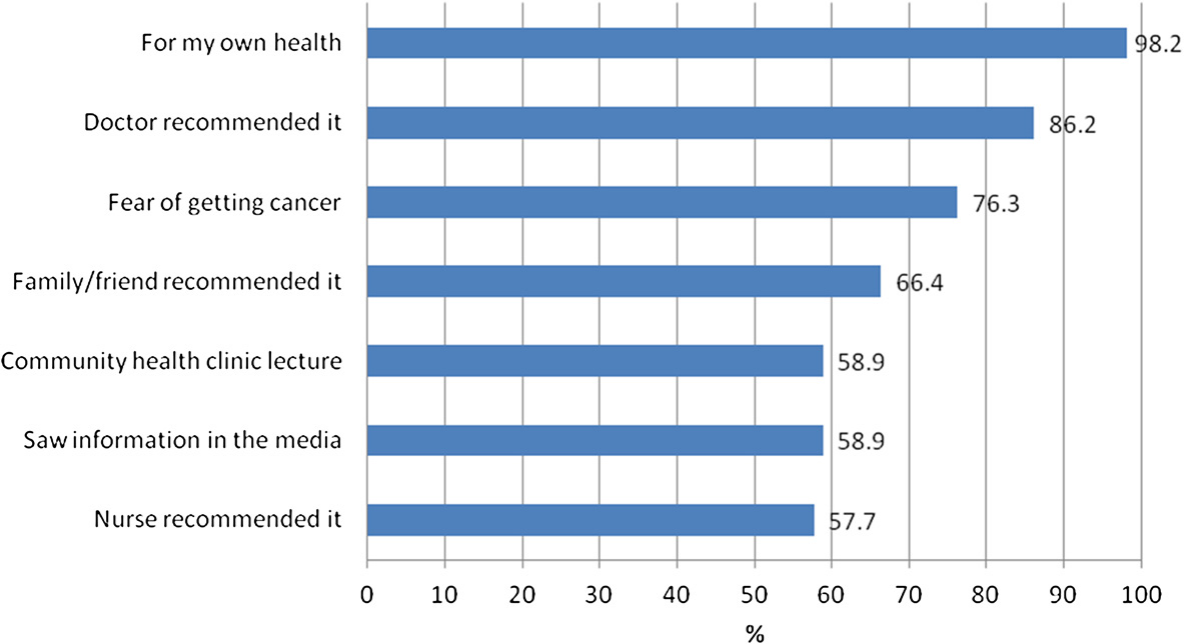
Reasons Participants are Planning Mammogram (40+ years, n = 333).

Of the participants who were planning to have a CBE (n = 525) or a mammogram (40 + years old, n = 333), over three quarters reported the following reasons: their own health, their doctor recommended it, or fear of getting cancer. Approximately two thirds said their plan to have a CBE or a mammogram was due to a recommendation from a family member/friend or nurse or from seeing information about breast cancer and its screening in the media or hearing about it in a community health clinic lecture (Figures 2 and 3).

**Figure 4:**
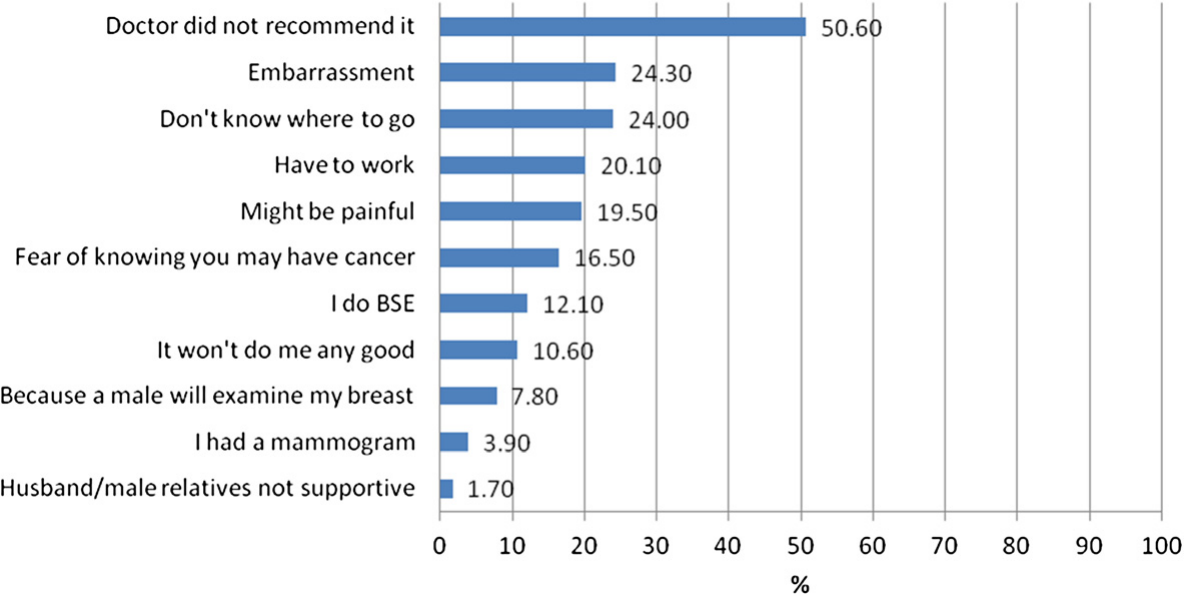
Reasons Participants are NOT Planning CBE (n = 538).

**Figure 5:**
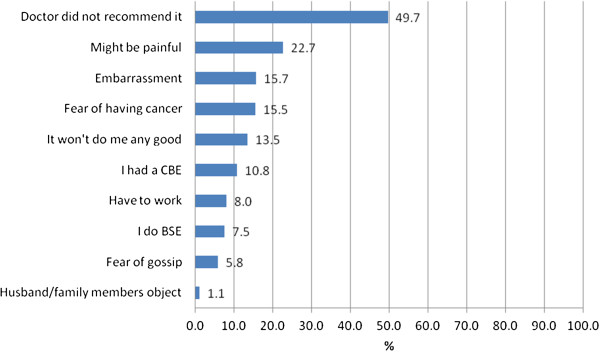
Reasons Participants are NOT Planning Mammogram (40+ years, n = 363).

The most common reason given by participants for not planning a CBE or mammogram (50.6% and 49.7%, respectively) was because their doctor did not recommend it. Approximately one fifth to one quarter of all participants said their failure to plan a CBE or a mammogram was due to embarrassment related to the procedure, not knowing where to go for a CBE or mammogram, possible pain involved in the mammogram procedure, or fear of cancer being discovered. Fewer participants reported reasons such as it would not do any good, a male might examine them, or fear of gossip. Less than 2% said their avoidance of CBE and mammography was due to their husbands’/male relatives’ disapproval of breast examinations (Figures 4 and 5).

### Associations among beliefs, attitudes, and BCS practice

Participants 40–49 years of age were significantly more likely to practice BSE or CBE (p < 0.05) than younger or older participants. Participants were more likely to practice BCS if they reported their health status to be good or excellent, if they thought that cancer is preventable, or if they thought that cancer is hereditary than participants who did not subscribe to these beliefs. Participants who stated they would want to know if they have cancer were more likely to have had or were planning to have a mammogram. Participants were significantly *less* likely to practice BCS if they believed cancer is God’s punishment or bad luck. Even though participants responded that cancer was due to fate, an unhealthy lifestyle, or not breastfeeding, these beliefs were not significantly related to BCS practice (Table 4).

**Table 4:** Beliefs, values and participation in breast cancer screening activities (n = 1063)

	**BSE practice**			**CBE practice**			**Mammogram practice**		
**Variables**	**Yes (%)**	**No (%)**		**Yes (%)**	**No (%)**		**Yes (%)**	**No (%)**	
	**n = 148**	**n = 915**	***P *** **value**	**n = 333**	**n = 730**	***P *** **value**	**n = 187**	**n = 508**	***P *** **value**
**Age (years)**			χ2(2, N = 1061) = 16.84, p < 0.001*			χ2(2, N = 1061) = 16.15, p < 0.001*			χ2(2, N = 695) = 0.21, p = 0.648
35 – 39	47 (12.9)	318 (87.1)		88 (24.1)	277 (75.9)		N/A	N/A	
40 – 49	75 (18.8)	324 (81.2)		150 (37.6)	249 (62.4)		110 (27.6)	289 (72.4)	
50+	24 (8.1)	273 (91.9)		94 (31.6)	203 (68.4)		77 (26.0)	219 (74.0)	
**Health status**			χ2(1, N = 1061) = 12.76, p < 0.001*			χ2(1, N = 1061) = 5.50, p = 0.019*			χ2(1, N = 694) = 5.72, p = 0.017*
Poor-Fair	18 (7.1)	234 (92.9)		64 (25.4)	188 (74.6)		44 (20.9)	167 (79.1)	
Good-Excellent	130 (16.1)	679 (83.9)		269 (33.3)	540 (66.7)		143 (29.6)	340 (70.4)	
**Can cancer be prevented?**			χ2(1, N = 1063) = 16.45, p < 0.001*			χ2(1, N = 1063) = 23.75, p < 0.001*			χ2(1, N = 695) = 13.44, p < 0.001*
No	62 (10.2)	546 (89.8)		154 (25.3)	454 (74.7)		87 (21.6)	315 (78.4)	
Yes	86 (18.9)	369 (81.1)		179 (39.3)	276 (60.7)		100 (34.1)	193 (65.9)	
**Participant would want to know if she had cancer**			χ2(1, N = 1063) = 0.95, p = 0.326			χ2(1, N = 1063) = 2.72, p = 0.099			χ2(1, N = 695) = 4.88, p = 0.027*
No	16 (11.3)	126 (88.7)		36 (25.4)	106 (74.6)		19 (18.1)	86 (81.9)	
Yes	132 (14.3)	789 (85.7)		297 (32.2)	624 (67.8)		168 (28.5)	422 (71.5)	
**Reasons participant believes people get cancer**									
**It’s God’s punishment**			χ2(1, N = 1063) = 3.91, p = 0.048*			χ2(1, N = 1063) = 14.05, p < 0.001*			χ2(1, N = 695) = 8.29, p = 0.004*
No	135 (14.8)	779 (85.2)		306 (33.5)	608 (66.5)		173 (28.8)	427 (71.2)	
Yes	13 (8.7)	136 (91.3)		27 (18.1)	122 (81.9)		14 (14.7)	81 (85.3)	
**It’s fate or destiny**			χ2(1, N = 1063) = 0.406, p = 0.524			χ2(1, N = 1063) = 0.39, p = 0.535			χ2(1, N = 695) = 0.81, p = 0.368
No	6 (17.6)	28 (82.4)		9 (26.5)	25 (73.5)		5 (19.2)	21 (80.8)	
Yes	142 (13.8)	887 (86.2)		324 (31.5)	705 (68.5)		182 (27.2)	487 (72.8)	
**It’s bad luck**			χ2(1, N = 1063) = 1.69, p = 0.194			χ2(1, N = 1063) = 4.29, p = 0.038*			χ2(1, N = 695) = 4.07, p = 0.044*
No	138 (14.4)	822 (85.6)		310 (32.3)	650 (67.7)		173 (28.1)	442 (71.9)	
Yes	10 (9.7)	93 (90.3)		23 (22.3)	80 (77.7)		14 (17.5)	66 (82.5)	
**Cancer is contagious**			χ2(1, N = 1063) = 0.06, p = 0.815			χ2(1, N = 1063) = 3.39, p = 0.066			χ2(1, N = 695) = 4.71, p = 0.030*
No	142 (14.0)	874 (86.0)		324 (31.9)	692 (68.1)		185 (27.6)	485 (72.4)	
Yes	6 (12.8)	41 (87.2)		9 (19.1)	38 (80.9)		2 (8.0)	23 (92.0)	
**Cancer is hereditary**			χ2(1, N = 1063) = 9.80, p = 0.002*			χ2(1, N = 1063) = 18.24, p < 0.001*			χ2(1, N = 695) = 13.07, p < 0.001*
No	32 (9.2)	317 (90.8)		79 (22.6)	270 (77.4)		49 (19.0)	209 (81.0)	
Yes	116 (16.2)	598 (83.8)		254 (35.6)	460 (64.4)		138 (31.6)	299 (68.4)	
**Unhealthy lifestyle**			χ2(1, N = 1063) = 0.16, p = 0.692			χ2(1, N = 1063) = 0.35, p = 0.553			χ2(1, N = 695) = 0.92, p = 0.338
No	11 (15.5)	60 (84.5)		20 (28.2)	51 (71.8)		19 (32.2)	40 (67.8)	
Yes	137 (13.8)	855 (86.2)		313 (31.6)	679 (68.4)		168 (26.4)	468 (73.6)	
**Not breastfeeding**			χ2(1, N = 1063) = 0.16, p = 0.204			χ2(1, N = 1063) = 0.64, p = 0.422			χ2(1, N = 695) = 0.28, p = 0.597
No	33 (16.8)	164 (83.2)		57 (28.9)	140 (71.1)		31 (25.0)	93 (75.0)	
Yes	115 (13.3)	751 (86.7)		276 (31.9)	590 (68.1)		156 (27.3)	415 (72.7)	

A higher education level of the participant or the participant’s husband was significantly related to a perceived good-excellent health status, the belief that cancer can be prevented, or the desire to know if cancer was present (p < 0.05). Participants with higher education levels were also more likely to believe that cancer can be hereditary or can arise from an unhealthy lifestyle, whereas less educated participants were more likely to believe that cancer is God’s punishment, bad luck, or contagious (p < 0.05).

### Attitudes toward health care, health care providers, and BCS practice

Participants whose doctors had talked to them about breast cancer and who understood the doctor’s message were significantly more likely to practice BCS. Participants who trusted their doctors and had no gender preference for their HCP were more likely to have had a CBE. Those who preferred to have a doctor rather than a nurse examine them were more likely to have a mammogram than those who preferred a nurse examine them or who had no preference. Language preference for one’s HCP (Arabic or English) was not significantly related to BCS practice (Table 5).

**Table 5:** Attitudes towards health care and health care providers

	**BSE practice**			**CBE practice**			**Mammogram practice**		
**Variables**	**Yes (%)**	**No (%)**		**Yes (%)**	**No (%)**		**Yes (%)**	**No (%)**	
	**n = 148**	**n = 915**	***P *** **value**	**n = 333**	**n = 730**	***P *** **value**	**n = 187**	**n = 508**	***P *** **value**
**Doctor talked to participant about breast cancer**			χ2(1, N = 1063) = 97.89, p < 0.001*			χ2(1, N = 1063) = 151.4, p < 0.001*			χ2(1, N = 695) = 63.22, p < 0.001*
No	64 (8.0)	740 (92.0)		172 (21.4)	632 (78.6)		102 (19.4)	425 (80.6)	
Yes	84 (32.4)	175 (67.6)		161 (62.2)	98 (37.8)		85 (50.6)	83 (49.4)	
**Participant understands doctor**			χ2(1, N = 1063) = 0.78, p = 0.377			χ2(1, N = 1063) = 37.67, p < 0.001*			χ2(1, N = 695) = 13.20, p < 0.001*
No	45 (12.6)	312 (87.4)		68 (19.0)	289 (81.0)		37 (17.6)	173 (82.4)	
Yes	103 (14.6)	603 (85.4)		265 (37.5)	441 (62.5)		150 (30.9)	335 (69.1)	
**Participant trusts doctor**									
No	23 (10.7)	191 (89.3)	χ2(1, N = 1063) = 2.25, p = 0.133	49 (22.9)	165 (77.1)	χ2(1, N = 1063) = 8.85, p = 0.003*	30 (21.7)	108 (78.3)	χ2(1, N = 695) = 2.34, p = 0.126
Yes	125 (14.7)	724 (85.3)		284 (33.5)	565 (66.5)		157 (28.2)	400 (71.8)	
**What kind of health care provider (HCP) would participant prefer to perform a breast examination?**									
Male or No preference	21 (21.4)	77 (78.6)	χ2(1, N = 1063) = 5.08, p = 0.024*	42 (42.9)	56 (57.1)	χ2(1, N = 1063) = 6.67, p = 0.010*	24 (33.8)	47 (66.2)	χ2(1, N = 695) = 1.91, p = 0.167
Female HCP	127 (13.2)	838 (86.8)		291 (30.2)	674 (69.8)		163 (26.1)	461 (73.9)	
Nurse or No preference	32 (10.9)	261 (89.1)	χ2(1, N = 1061) = 2.75, p = 0.097	81 (27.6)	212 (72.4)	χ2(1, N = 1061) = 2.50, p = 0.114	42 (20.2)	166 (79.8)	χ2(1, N = 694) = 6.61, p = 0.010*
Doctor	114 (14.8)	654 (85.2)		251 (32.7)	517 (67.3)		144 (29.6)	342 (70.4)	
Non-Arab or no preference	73 (14.1)	444 (85.9)	χ2(1, N = 1063) = 0.03, p = 0.857	162 (31.3)	355(68.7)	χ2(1, N = 1063) = 0.00, p = 0.996	88 (27.7)	230 (72.3)	χ2(1, N = 695) = 0.18, p = 0.676
Arab HCP	75 (13.7)	471 (86.3)		171 (31.3)	375 (68.7)		99 (26.3)	278 (73.7)	

Further analyses on age and education differences indicated that older participants (50+ years) and those with lower education levels were more likely to have no preference for the status of HCP (doctor or nurse) that examined them, did not want to know if they had cancer, and believed cancer result from not breastfeeding (p < 0.05).

### Beliefs and attitudes related to CBE and mammogram practice

Participants who were planning to have a CBE “for health” were significantly more likely to have had a CBE. Participants were less likely to have had a CBE and less likely to plan to have a future CBE if they perceived a CBE might be painful/ uncomfortable, if they were afraid cancer might be discovered, or they felt the procedure would embarrass them (p < 0.05) (Table 6).

**Table 6:** Reasons for planning CBE or non-compliance and CBE practice

	**CBE practice**		
**Variables**	**Yes (%)**	**No (%)**	***P *- value**
**Reasons participants planned CBE (n = 525)**			
For her own health			χ2(1, N = 525) = 4.74, p = 0.030*
No	2 (14.3)	12 (85.7)	
Yes	222 (43.4)	289 (56.6)	
Fear of getting cancer as a reason for CBE			χ2(1, N = 525) = 2.57, p = 0.109
No	46 (36.5)	80 (63.5)	
Yes	178 (44.6)	221 (55.4)	
**Reasons participants did NOT plan CBE (n = 538)**			
Might be painful or uncomfortable			χ2(1, N = 538) = 11.03, p = 0.001*
No	100 (23.1)	333 (76.9)	
Yes	9 (8.6)	96 (91.4)	
Fear of knowing you might have cancer			χ2(1, N = 538) = 8.39, p = 0.004*
No	101 (22.5)	348 (77.5)	
Yes	8 (9.0)	81 (91.0)	
Embarrassment			χ2(1, N = 538) = 19.22, p < 0.001*
No	100 (24.6)	307 (75.4)	
Yes	9 (6.9)	122 (93.1)	
It won’t do her any good			χ2(1, N = 538) = 2.51, p = 0.113
No	102 (21.2)	379 (78.8)	
Yes	7 (12.3)	50 (87.7)	
Because a male will examine her breasts			χ2(1, N = 538) = 3.25, p = 0.071
No	105 (21.2)	391 (78.8)	
Yes	4 (9.5)	38 (90.5)	
Husband or male relatives not supportive			χ2(1, N = 538) = 0.47, p = 0.491
No	108 (20.4)	421 (79.6)	
Yes	1 (11.1)	8 (88.9)	

Participants with lower education levels were more likely than those with higher education levels (p < 0.05) to give the following reasons for not planning a CBE: fear of finding cancer, and the belief that a CBE would not be beneficial.

Participants 40 years of age or older were significantly less likely to have had a mammogram if they stated the following reasons for not planning a future mammogram: a mammogram might be painful/uncomfortable, fear of knowing they might have cancer, fear of gossip, or embarrassment (p < 0.05) (Table 7).

**Table 7:** Reasons for planning mammogram or non-compliance and mammogram practice (40+ years old)

	**Mammogram practice**		
**Variables**	**Yes (%)**	**No (%)**	*P *– value*
**Reasons participants planned mammogram (n = 332)**			
For her own health			χ2(1, N = 332) = 3.60, p = 0.058
No	0 (0.0)	6 (100.0)	
Yes	123 (37.7)	203 (62.3)	
Fear of getting cancer as a reason for mammogram			χ2(1, N = 332) = 0.01, p = 0.943
No	29 (36.7)	50 (63.3)	
Yes	94 (37.2)	159 (62.8)	
**Reasons participants did NOT plan mammogram (n = 363)**			
Might be painful or uncomfortable			χ2(1, N = 363) = 4.52, p = 0.033*
No	56 (19.9)	225 (80.1)	
Yes	8 (9.8)	74 (90.2)	
Fear of knowing you might have cancer			χ2(1, N = 363) = 11.45, p = 0.001*
No	63 (20.5)	244 (79.5)	
Yes	1 (1.8)	55 (98.2)	
Fear of gossip			χ2(1, N = 363) = 4.77, p = 0.029*
No	64 (18.7)	278 (81.3)	
Yes	0 (0.0)	21 (100.0)	
Embarrassment			χ2(1, N = 363) = 14.47, p < 0.001*
No	64 (20.9)	242 (79.1)	
Yes	0 (0.0)	57 (100.0)	
It won’t do her any good			χ2(1, N = 363) = 0.44, p = 0.509
No	57 (18.2)	257 (81.8)	
Yes	7 (14.3)	42 (85.7)	
Husband or male relatives not supportive			χ2(1, N = 363) = 0.87, p = 0.352
No	64 (17.8)	295 (82.2)	
Yes	0 (0.0)	4 (100.0)	

*Significant at 0.05 level.

Mammogram practice was not significantly related to participants’ perceptions that a mammogram would not have benefits. However, older participants were more likely to state that their doctors did not recommend having a mammogram, that a mammogram would not do any good, or that they were afraid that cancer might be discovered (p < 0.05). It is important to note that nonsupport of husband or male relatives was not significantly related to participants’ intention to avoid mammography.

### Multivariate analysis of factors associated with BCS practice

Table 1 represents the multivariate logistic regression analysis of beliefs and attitudes that were significantly associated with BCS practice.

### BSE practice

Participants who believed their health status was good–excellent (OR = 2.03; 95% CI = 1.20 – 3.44; p = 0.009), that cancer could be prevented (OR = 1.85; 95% CI = 1.29 – 2.67; p = 0.001), or that cancer is hereditary (OR = 1.68; 95% CI = 1.09 – 2.57; p = 0.018) were twice as likely to practice BSE than those who did not subscribe to these beliefs. Participants who preferred female rather than male HCPs to examine them were half as likely to practice BSE (OR = 0.49; 95% CI = 0.29 – 0.85; p = 0.010) as those who did not care whether the examining HCP was male or female.

### CBE practice

Participants who believed cancer could be prevented (OR = 1.59; 95% CI = 1.21 – 2.10; p = 0.001), who understood their doctor’ message (OR = 2.15; 95% CI = 1.55 – 2.98; p < 0.001), and believed cancer is hereditary (OR = 1.73; 95% CI = 1.27 – 2.36; p = 0.001) were more likely to have had a CBE than those who did not. Participants who believed that cancer is God’s punishment (OR = 0.52; 95% CI = 0.33 – 0.83; p = 0.006) or preferred a female HCP rather than a male HCP to examine them (OR = 0.56; 95% CI = 0.36 – 0.88; p = 0.011) were approximately half as likely to have had a CBE than those who did not believe cancer is God’s punishment or did not care whether the examining HCP was male or female.

Predictors for not planning a CBE included participants’ fear of knowing they might have cancer (OR = 0.43; 95% CI = 0.20 – 0.94; p = 0.034) and participants’ embarrassment regarding the CBE procedure (OR = 0.32; 95% CI = 0.15 – 0.70; p = 0.004).

### Mammogram practice

Participants (40+ years of age) were more likely to have had a mammogram if they understood their doctor’s message (OR = 1.81; 95% CI = 1.18 – 2.79; p = 0.007), if they believed cancer could be prevented (OR = 1.59; 95% CI = 1.11 – 2.26; p = 0.011), or if they believed cancer is hereditary (OR = 1.68; 95% CI = 1.14 – 2.48; p = 0.009). Participants were half as likely to have had a mammogram if they believed that cancer is God’s punishment (OR = 0.51; 95% CI = 0.28 – 0.95; p = 0.035) than those who did not have this belief.

Fear of knowing one might have cancer (OR = 0.09; 95% CI = 0.01– 0.69; p = 0.021) was a significant predictor for not planning to have a mammogram.

## Discussion

These findings provide a partial answer to the question of why, despite the availability of health care services and gender-appropriate health care providers in Qatar, less than one third of the women interviewed practiced BCS according to national guidelines. Consistent with Kleinman’s explanatory model of health, illness and disease, this study found that several complex beliefs and attitudes toward breast cancer screening influence BCS uptake among Arabic-speaking women in Qatar. As with Arab or Muslim women living in the West, the women interviewed had low BCS awareness and low screening rates, they overwhelmingly preferred female physicians, and they gave fear or embarrassment as reasons for why they did not plan to have a CBE or mammogram [40,41]. However, the majority of participants wanted to know if they have cancer, would make a mammogram appointment if advised to, and trusted their physicians. These encouraging findings indicate that Qatari women’s fears of cancer and their low participation in cancer screening could be effectively addressed with culturally appropriate awareness and intervention programs.

It is important to note that fear of cancer is apparently both a facilitator and a barrier to BCS participation, a finding congruent with previous studies in the region [9,15,17,30]. It is likely that the fear of knowing one might have cancer is related to the cultural impact it would have on a woman’s life or her family’s dynamics, and the belief that a cancer diagnosis is a death sentence [6]. When beliefs such as fate and fear of cancer are blended with cancer fatalism, they can act as significant barriers to BCS [6,26,42]. Evidence that fatalism is a barrier to health care practice in the Middle East remains inconclusive; exploring what a cancer diagnosis means to an Arab woman and how it impacts her life requires further research [6,42].

Previous studies indicate that low perceived risk and pessimistic views related to cancer among Arab women can act as barriers to cancer treatment or BCS [9,15,43,44]. In the current study, women whose self-perceived health status was good–excellent, who believed cancer is preventable and that cancer might be hereditary were more likely to practice BCS, indicating that higher perceived risk can lead to greater screening practices among Arab women. Participants with lower education levels were more likely than women with higher education levels to not plan a CBE because they feared that cancer might be discovered or believed that screening is not beneficial. Thus, public educational campaigns should be more inclusive of groups or individuals with lower levels of education. As breast cancer can be asymptomatic in early stages, it may be difficult for women to perceive their risk. Educational materials on BCS should clearly point out that breast cancer screening can increase survival rates and treatment options when diagnosed early, in languages that women in Qatar can understand.

Arab patients greatly respect their physicians as experts whose advice is followed [45]. While our study concurs with previous findings that doctor recommendations facilitate BCS practices among Arab women [9,26], only one quarter of the women we interviewed said their doctors had talked to them about breast cancer. It has been argued that effective communication between physician and patient must address culturally-sensitive concerns to adequately influence decision making and health seeking behaviors of patients [32,33,46]. Because physician-initiated discussion about breast cancer was the strongest predictor for BCS, it is imperative that conversations about breast cancer and early detection be routinely discussed during patient visits to health centers in Qatar. Many women in Qatar go to see doctors at community health clinics only when they or their family members are ill, therefore, physician-initiated discussions about breast cancer screening are essential during these visits as it might be the only opportunity for these women to be educated about breast cancer. Although women can call the mammogram clinic for screening appointment, given the very low utilization of CBE and mammograms, physicians and other health care professionals’ explanations of the benefit of BCS and how a mammogram can save a woman’s life, would facilitate women’s willingness overcome barriers to screening.

Most Qatari women are Muslim and report having a strong faith in God [16]. Modesty, taking care of one’s health, and a belief in fate are part of the belief structures in Islam. Whereas many women interviewed believed that getting cancer is due to fate, our analyses did not indicate that this belief was an indicator for a passive approach to cancer or BCS. Modesty is considered a virtue in Islam; however, religious guidelines allow Muslims to have their bodies examined by health care professionals for medical reasons. Nevertheless, modesty and embarrassment clearly hinder some women from having their breasts examined by health care professionals, especially by male HCPs [15,17,29,47,48], or causes them to seek care only when symptoms worsen [49,50]. Although less than 2% of the women interviewed stated that their husbands or male relatives objected to breast examination, their preference for female HCPs was significantly related to having had a CBE. Because the State of Qatar provides gender-appropriate HCPs in most hospitals and health centers, concerns about HCP gender may be a perceived barrier that can be alleviated with greater awareness of health care services that are culturally and religiously compliant with most Arab women’s beliefs. Including religious leaders as part of awareness campaigns aimed at promoting BCS as being congruent with Islamic principles should be an essential part of a culturally appropriate intervention strategy, and has been found to increase the rate of awareness and success of intervention programs for Arab or Muslim women [40,41]. Arab breast cancer survivors can also play an important role in communicating that breast cancer is a chronic disease rather than a fatal one. The goals of awareness campaigns should be to lessen fears and stigma and encourage women to participate in screening activities.

It is difficult to generalize the results of this study to all women living in Qatar because of the non-probability convenience sampling. An attempt to increase generalizability and to reduce potential bias was made by randomly-selecting times to reach every potential participant who met study’s inclusion criteria at all research sites, resulting in a high response rate of 87.5%. Also, data were collected from self-reported interviews, which might be affected by recall or social-desirability response bias. However, the results of this study give insights into breast cancer screening practices of Qatari women that can be applied to women with similar sociocultural backgrounds throughout the Middle East and globally.

## Conclusion

This study’s findings indicate low levels of awareness of BCS and low participation rates in BCS among women in Qatar. Women who engaged in BCS practice were more likely to have a doctor who talked to them about breast cancer, to believe they were in good–excellent health, to believe that cancer can be prevented, or to believe that cancer might be hereditary. While the majority of participants stated they would want to know if they had cancer and felt their health care needs were being met, their main reasons for not planning BCS were lack of a doctor’s recommendation, fear of being diagnosed with cancer, fear of possible discomfort in the BCS procedures, and embarrassment in undergoing BCS.

As women in Qatar become more educated and aware of health and disease, health care providers and policy makers must facilitate Arab women’s desire to know more about the benefits of cancer screening for early detection of this disease. Similar to the findings of a qualitative study of Iraqi women living in the U.S. [51], the majority of women living in Qatar are very responsive to the message promoting breast cancer screening and are eager to participate in its screening activities. Caring for one’s health is ingrained in many women’s belief systems, and can be used to promote breast cancer screening as a religious duty of both women and men to do what is beneficial for themselves and their families. A well known prophetic saying in Islam is: “Your body has a right over you” [52].

If female and male health care professionals would make breast cancer and its screening a mandatory topic of discussion with all gender and age-appropriate women and men, awareness could be raised of culturally-appropriate services available in Qatar. Public health awareness campaigns that highlight these services and regular physician-patient discussions about BCS can be cost-effective and efficient strategies to integrate BCS into the public consciousness while effort is being made to advance national cancer registries and national population based screening programs in Qatar.

Ensuring ongoing screening practices requires culturally appropriate community support from respected elders, religious leaders, and breast cancer survivors. Collaboration between researchers, community leaders, health care professionals, and policy makers is important to ensure the appropriateness and success of educational and outreach campaigns aimed at increasing screening uptake and reducing morbidity and mortality related to breast cancer among women in Qatar.

## Abbreviations

BCS: Breast cancer screening; BSE: Breast self examination; CBE: Clinical breast examination; HCP: Health care provider.

## Competing interests

The authors declare that they do not have any competing interests.

## Authors’ contributions

DT: Contributed to the conception and design of the study and the acquisition, analysis, and interpretation of data, drafted the manuscript, and gave final approval of the manuscript version submitted for publication. AKA: Contributed to the conception and design of the study and the acquisition of data, revised the manuscript, and gave final approval of the manuscript version submitted for publication. BAS: Contributed to the conception and design of the study and the acquisition of data, revised the manuscript, and gave final approval of the manuscript version submitted for publication. AKM: Contributed to the conception and design of the study and the acquisition of data, reviewed the manuscript critically for content, and gave final approval of the manuscript version submitted for publication. AMN: Contributed to the conception and design of the study and the acquisition of data, revised the manuscript, and gave final approval of the manuscript version submitted for publication. MM: Contributed to the conception and design of the study and the acquisition of data, reviewed the manuscript critically for content, and gave final approval of the manuscript version submitted for publication. SR: Contributed to the conception and design of the study and the acquisition, analysis, and interpretation of data, revised the manuscript, and gave final approval of the manuscript version submitted for publication. CS: Contributed to the analysis and interpretation of data, drafted the manuscript, and gave final approval of the manuscript version submitted for publication. FT: Contributed to the analysis and interpretation of data, revised the manuscript, and gave final approval of the manuscript version submitted for publication. All authors read and approved the final manuscript.

## Pre-publication history

The pre-publication history for this paper can be accessed here:

http://www.biomedcentral.com/1472-6874/13/49/prepub

## A note on study terminology

“Arabic women” is used to refer to female Arabic speakers, despite the fact that they may not be ethnically Arab. A participant who is “aware” has an understanding of breast cancer and its screening recommendations according to current national screening guidelines. A participant who “practices BCS” engages in BCS activities according to current recommended guidelines.
